# Molecular profiling supports the role of epithelial-to-mesenchymal transition (EMT) in ovarian cancer metastasis

**DOI:** 10.1186/1757-2215-6-49

**Published:** 2013-07-10

**Authors:** Loukia N Lili, Lilya V Matyunina, L DeEtte Walker, Stephen L Wells, Benedict B Benigno, John F McDonald

**Affiliations:** 1 Integrated Cancer Research Center, School of Biology, and Parker H. Petit Institute of Bioengineering and Biosciences, Georgia Institute of Technology, 315 Ferst Dr., Atlanta, GA 30332, USA; 2 Northside Hospital, 1000 Johnson Ferry Rd, Atlanta, GA 30342, USA; 3 Ovarian Cancer Institute, 960 Johnson Ferry Rd, Suite 130, Atlanta GA 30342, USA

**Keywords:** Metastasis, Epithelial-to-mesenchymal transition, EMT, Ovarian cancer

## Abstract

**Background:**

While metastasis ranks among the most lethal of all cancer-associated processes, on the molecular level, it remains one of the least well understood. One model that has gained credibility in recent years is that metastasizing cells at least partially recapitulate the developmental process of epithelial-to-mesenchymal transition (EMT) in their transit from primary to metastatic sites. While experimentally supported by cell culture and animal model studies, the lack of unambiguous confirmatory evidence in cancer patients has led to persistent challenges to the model’s relevance in humans.

**Methods:**

Gene expression profiling (Affymetrix, U133) was carried out on 14 matched sets of primary (ovary) and metastatic (omentum) ovarian cancer (serous adenocarcinoma) patient samples. Hierarchical clustering and functional pathway algorithms were used in the data analysis.

**Results:**

While histological examination reveled no morphological distinction between the matched sets of primary and metastatic samples, gene expression profiling clearly distinguished two classes of metastatic samples. One class displayed expression patterns statistically indistinguishable from primary samples isolated from the same patients while a second class displayed expression patterns significantly different from primary samples. Further analyses focusing on genes previously associated with EMT clearly distinguished the primary from metastatic samples in all but one patient.

**Conclusion:**

Our results are consistent with a role of EMT in most if not all ovarian cancer metastases and demonstrate that identical morphologies between primary and metastatic cancer samples is insufficient evidence to negate a role of EMT in the metastatic process.

## Background

Epithelial-to-mesenchymal transition (EMT) is a process by which epithelial cells acquire mesenchymal cell characteristics including reduced cell adhesiveness and increased cell motility [1]. The process is an essential component of embryonic development and is known to be both transient and reversible (mesenchymal-to-epithelial transition or MET). Initial observations that many characteristics of cancer metastasis appear to be recapitulations of key features of EMT/MET, led to the hypothesis that similar, if not identical, molecular mechanisms may be involved [2-4]. While numerous subsequent studies conducted in cell lines and animal models have supported this hypothesis (e.g., [5-8]), the lack of unambiguous evidence from studies involving human tumor samples has resulted in skepticism [9,10]. One persistent objection to the model is the fact that careful morphological examinations of human metastases have never uncovered the existence of cancer mesenchymal-like cells [9]. Indeed, while it is clear that cancer metastasis must involve detachment of cancer cells from the primary tumor, such a phenomenon could well be attributed to mutation(s) and/or aberrant gene expression patterns in one or a few genes and not necessarily reflect involvement of a more coordinated process such as EMT [9]. Although these alternative possibilities are not mutually exclusive, a clarification of the molecular mechanism(s) underlying metastasis is critical because it could have significant ramifications on future directions in the development of diagnostic tests and potential anti-metastatic drugs [11].

Ovarian cancer is the most malignant of all gynecological cancers and is responsible for over 14,000 deaths per year in the United States alone [12]. Because ovarian cancer is often asymptomatic early in its progression (Stages I/II), the disease is not typically diagnosed until later stages (Stages III/IV) when the cancer has metastasized and prognosis is poor (5 year survival < 30%) [12]. Ovarian cancer metastasis is primarily due to the exfoliation of malignant cells or cell aggregates from the primary tumor into the abdominal cavity and their subsequent spread and attachment to visceral and parietal peritoneal surfaces of abdominal organs such as the omentum. This mechanism of intra-abdominal metastatic spread allows for the capture and molecular comparison of primary and metastatic cancer cells isolated from the same patient, providing a favorable opportunity to evaluate the potential role of epithelial-to-mesenchymal transition (EMT) in the metastatic process.

## Methods

### Tissue collection

Tissues were collected at Northside Hospital (Atlanta, GA) under appropriate Institutional Review Board protocols (H09227). Following resection, tissues were histologically examined samples placed in cryotubes and immediately (<1 minute) frozen in liquid nitrogen. Samples were transported on dry ice to Georgia Institute of Technology (Atlanta, GA), and stored at −80°C. After examination and verification by a pathologist, tissues were embedded in cryomatrix (Shandon). The clinical information of the primary and metastatic cancer tissues from the seven patients is found in Additional file 1.

For each of the primary and metastatic (omental) tissue samples, 7 μm frozen sections were cut and attached to uncharged microscope slides. Immediately following dehydration and staining (HistoGene, LCM Frozen Section Staining Kit, Arcturus. Life Technologies), slides were processed in an Autopix (Arcturus) instrument for laser capture microdissection (LCM). CapSure Macro-LCM Caps (Arcturus, Life Technologies) were used to ensure purity of all primary cancer epithelial cells and omental metastatic cells. Approximately 30,000 cells were collected for each of the 14 tissue samples (seven primary and seven matched metastatic ovarian cancer samples).

### RNA extraction and amplification

PicoPure RNA Isolation Kit (Life Technologies) protocols were followed for RNA extraction from the cells on the Macro-LCM caps in 30 μL of extraction buffer. RNA quality was verified for all samples on the Bioanalyzer RNA Pico Chip (Agilent Technologies). Total RNA from the above extractions was processed using the Ovation® Pico WTA System (NuGEN) in conjunction with the Encore™ BiotinIL Module (NuGEN), to produce an amplified, biotin-labeled cDNA suitable for hybridizing to GeneChip Human Genome U133 Plus 2.0 Arrays (Affymetrix) following manufacturer’s recommendations.

### Microarray data analysis

Fourteen individual gene expression profiles were generated from the primary and matched metastatic samples of each of the seven patients used in this study. The 14 Affymetrix .CEL files were processed using the Affymetrix Expression Console (EC) Software Version 1.1 using the Robust Multi-Array Average (RMA) normalization method. The normalized expression values from all 14 samples were log_2_ transformed. From the initial log_2_ transformed 54,675 probe sets (21,049 probe sets transformed, unique, annotated genes), 50,286 that displayed marginal differences in expression across all patient samples (standard deviation ≤ 0.8 from the mean of all 14 samples) were filtered out. The remaining 4,389 probe sets (3,365 genes) were employed in the unsupervised clustering analysis.

For the identification of the epithelial to mesenchymal transition (EMT) related genes, a list of 84 genes, previously implicated in the process of EMT, was employed (http://www.sabiosciences.com/rt_pcr_product/HTML/PAHS-090Z.html). Of these genes, 39 (61 probe sets) were identified among the 3,365 genes (4,389 probe sets) differentially expressed across samples.

Individual clustering analyses were carried out for matched sets of primary and metastatic samples from the Group 1 and Group 2 patients. All hierarchical clustering was performed using normalized Z-scores of the log_2_ transformed expression values.

### Functional pathway enrichment

Biological interpretations of the differential gene expression data were performed by pathway enrichment analysis using MetaCore 5.2 (GeneGO, St Joseph, MI, http://thomsonreuters.com/metacore/).

## Results and discussion

Fourteen matched sets of primary and metastatic (omental) samples were collected from seven advanced staged (III/IV) ovarian cancer (serous adenocarcinoma) patients. Pathological examination classified all of the cancer samples as highly un-differentiated with no significant difference in morphology between any of the primary and metastatic sets (Additional file 1). Tissue samples for molecular analysis were snap frozen in liquid nitrogen within one minute of surgical removal and subsequently embedded in cryomatrix (Shandon) for frozen sectioning and laser capture microdissection (LCM). Approximately 30,000 cancer epithelial cells were isolated from each tissue sample and RNA was extracted and processed for gene expression analysis (Affymetrix, U133Plus 2.0 arrays) according to previously described methods [13].

**Figure 1:**
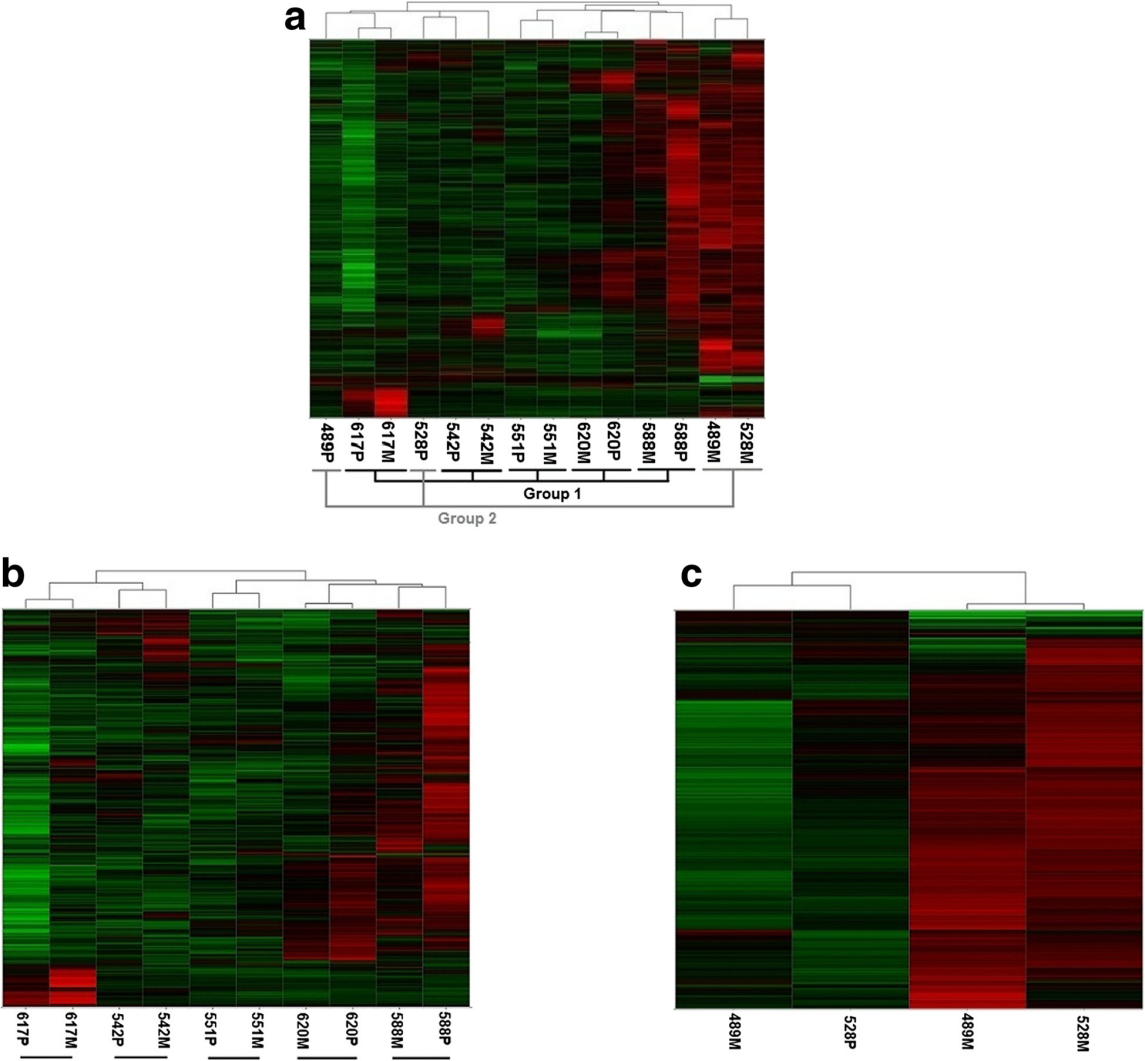
**Unsupervised classification of differentially expressed genes between primary and metastatic samples identifies two groups of patients. (a)** Unsupervised hierarchical clustering performed on 3,365 unique, annotated genes (4,389 probe sets) displaying significant expression variation across all samples (SD ≥ 0.8). Primary and metastatic samples from 5 patients (617, 542, 551, 620, 588) clustered closely to one another (Group1) while primary and metastatic samples from 2 patients (489, 528) clustered distantly from one another (Group 2); **(b)** Unsupervised hierarchical clustering of the same genes/probe sets in (a) across primary (P) and metastatic (M) samples of Group 1 patients. All P samples cluster most closely with their matched M samples for all patients; **(c)** Unsupervised hierarchical clustering of the same genes/probe sets in (a) across primary (P) and metastatic (M) samples of Group 2 patients. The P samples do not cluster with the matched M samples of the same patient.

Analysis of expression profiles for the 14 primary and metastatic samples identified 3,365 genes (4,389 probe sets) as being significantly differentially expressed (Additional file 2). Unsupervised hierarchical clustering of these data revealed that the metastatic samples of five of the seven patients grouped closely with their respective primary samples (Figure 1a, b, Group 1). In contrast, the metastatic samples from two of the patients (489 M and 528 M) clustered most closely with one another and distant from their respective primary samples (Figure 1a,c, Group 2). Functional pathway analysis was carried out incorporating the 3,365 differentially expressed genes described above. The results indicated that 13 of the 20 most significantly enriched pathways are associated with EMT or EMT-related functions (*i.e*., cytoskeleton remodeling, cell adhesion, *etc*., Table 1).

**Table 1:** **The 20 most significantly enriched pathways between all primary and metastatic samples**.

	**Functional pathways**	**pValue**
1	**Cytoskeleton remodeling_TGF, WNT and cytoskeletal remodeling**	1.53E-13
2	**Cell adhesion_ECM remodeling**	1.03E-11
3	**Cytoskeleton remodeling_Cytoskeleton remodeling**	7.54E-11
4	**Development_Regulation of epithelial-to-mesenchymal transition (EMT)**	9.43E-11
5	**Cell adhesion_Chemokines and adhesion**	1.75E-10
6	Oxidative phosphorylation	1.99E-10
7	**Development_TGF-beta-dependent induction of EMT via MAPK**	2.05E-09
8	DNA damage_Brca1 as a transcription regulator	3.36E-09
9	**Cytoskeleton remodeling_Reverse signaling by ephrin B**	6.64E-09
10	LRRK2 in neurons in Parkinson’s disease	2.32E-08
11	Apoptosis and survival_BAD phosphorylation	5.22E-08
12	**Development_TGF-beta-dependent induction of EMT via RhoA, PI3K and ILK**	5.30E-08
13	**Cytoskeleton remodeling_Role of PKA in cytoskeleton reorganisation**	1.30E-07
14	Transcription_Androgen Receptor nuclear signaling	2.02E-07
15	**Immune response_MIF-induced cell adhesion, migration and angiogenesis**	3.06E-07
16	Immune response_MIF - the neuroendocrine-macrophage connector	3.06E-07
17	**Development_TGF-beta-dependent induction of EMT via SMADs**	5.02E-07
18	**Cell adhesion_Integrin-mediated cell adhesion and migration**	6.77E-07
19	Transport_Clathrin-coated vesicle cycle	7.47E-07
20	**Cell adhesion_Role of tetraspanins in the integrin-mediated cell adhesion**	1.27E-06

Enriched pathways were computed utilizing the 3,365 significantly differentiated expressed genes (4,389 probe sets) represented in the clustering analysis (Figure 1). Thirteen of the 20 pathways (highlighted in **bold)** are involved in EMT or EMT-related processes (*iCe*., cytoskeleton remodeling, cell adhesion, EMT developmental processes, MIF-associated cell adhesion).

**Figure 2:**
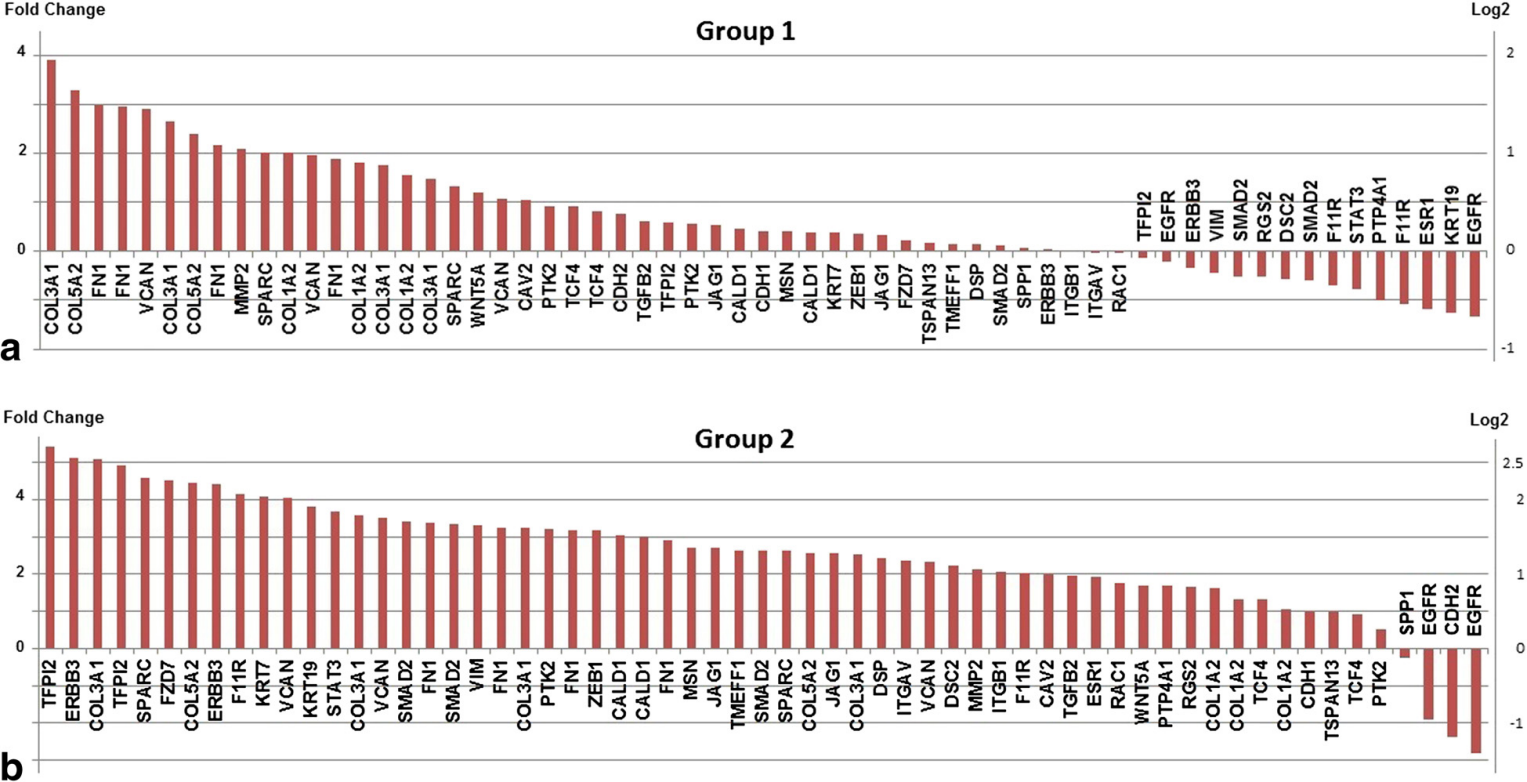
**Comparative ranking of EMT-associated genes with respect to fold-change differences in expression between the Group 1 and Group 2 primary and metastatic samples.** Thirty-nine previously characterized EMT associated genes were identified among the 3,365 genes significantly differentially expressed across 14 tissue samples (see Figure 1). Histograms depict the fold change differences in expression between primary and metastatic samples of Group 1 **(panel a)** and Group 2 **(panel b)** patients. Although EMT-associated genes are more differentially expressed between primary and metastatic samples from Group 2 than Group 1 patients, Group 1 patients also display large fold differences for some EMT-associated genes. These findings suggest that the observed differences in expression of EMT-associated genes between the primary and metastatic samples of Group 1 vs. Group 2 patients represent differences in a continuum of the EMT-MET process rather than its occurrence in one and absence in the other.

To explore the possibility that differences in the expression of EMT-associated genes may contribute to the dichotomy between Group 2 primary and metastatic samples, we conducted additional analyses focusing on genes previously established to be directly or indirectly involved in the EMT process (Additional file 3). Thirty-nine of these EMT-associated genes (61 probe sets) are among the 3,365 genes (4,389 probe sets) used in our clustering analysis (Figure 1). Figure 2 presents a comparative ranking of these 39 EMT-associated genes with respect to fold-change differences in expression between the Group 1 and Group 2 primary and metastatic samples. Nearly 74% (45/61) of the EMT-associated probe sets display a > 2-fold change in expression in the Group 2 metastatic samples while only 18% (11/61) display a > 2-fold difference in expression in the Group 1 metastatic samples. In addition, a number of the differences in expression are consistent with the hypothesis that, on the molecular level, Group 2 metastatic samples are more mesenchymal-like than their matched primary samples. For example, the expression of several “mesenchymal biomarkers”, *i.e.*, VIM (vimentin), TMEFF1 (transmembrane protein with EGF-like and two follistatin-like domains 1), ITGAV (integrin alpha-V), and ZEB1 (zinc finger E-box-binding homeobox1) are all up regulated (2 to 3-fold) in Group 2 metastatic samples while being essentially unchanged between Group 1 primary and metastatic samples. However, a number of other genes typically up-regulated during EMT [*e.g*., COL3A1 (collagen type III alpha-1), FN1 (fibronectin), VCAN (vesican), and MMP2 (matrix metalloproteinase-2] were not only up-regulated in Group 2 metastatic samples but in Group 1 metastatic samples as well, albeit at generally lower levels. These findings suggest that the observed differences in expression of EMT-associated genes between the primary and metastatic samples of Group 1 vs. Group 2 patients represent differences in a continuum of the EMT-MET process rather than its occurrence in one and absence in the other.

**Figure 3:**
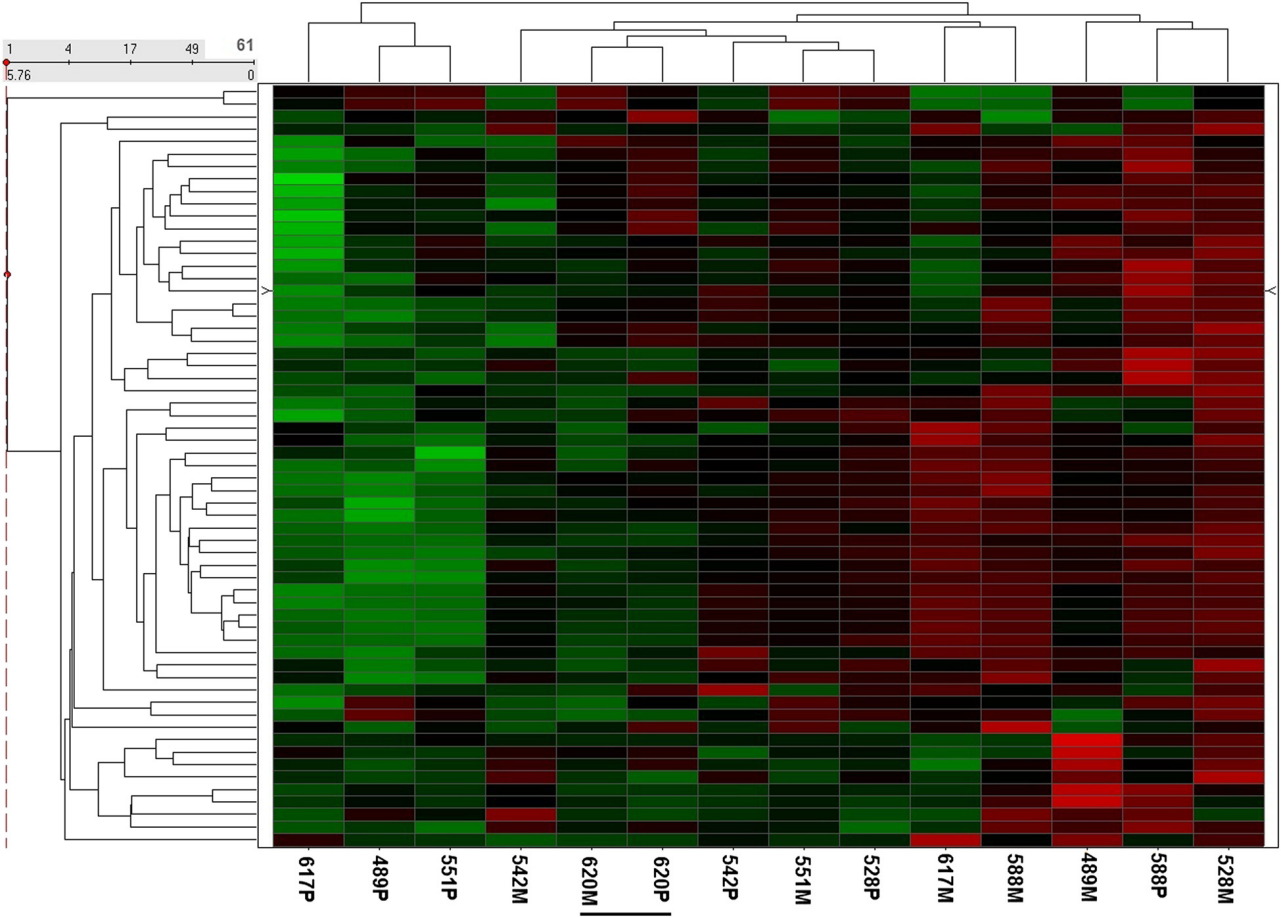
**Unsupervised classification of 39 differentially expressed EMT associated genes (61 probe sets) demonstrates significant divergence between most primary and metastatic samples.** Unsupervised hierarchical clustering of EMT associated genes differentially expressed across all samples demonstrates that the primary and metastatic samples of only one patient (620) are clustered most closely with one another. Primary and metastatic samples of all other patients cluster away from one another consistent with a model whereby all of the metastatic samples have undergone EMT while displaying a range of partial or complete (patient 620) compensating MET transitions at the metastatic site. The alternative hypothesis that metastasis occurs in the absence of EMT is definitively consistent with the molecular profiles of only one Group 1 patient (620).

To explore this possibility further, we conducted a second clustering analysis using only genes previously implicated in EMT (Additional file 3). The results (Additional file 4) presented in Figure 3 demonstrate that with respect to these EMT-associated genes, the primary sample of only one patient (620) remained most closely clustered with its metastatic counterpart. The primary and metastatic samples of all of the remaining patients displayed various degrees of isolation from one another. Collectively, our results are consistent with a model whereby all of the metastatic samples have undergone EMT while displaying a range of partial or complete (patient 620) compensating MET transitions at the metastatic site. The alternative hypothesis that metastasis occurs in the absence of EMT is definitively consistent with the molecular profiles of only one Group 1 patient (620).

The majority of cancer-related deaths are attributable to metastases rather than to primary tumors [14]. For this reason, there is considerable interest in understanding the molecular mechanisms underlying the process with the hope that such knowledge may lead to more effective therapeutic treatments [11]. Considerable evidence has accumulated in recent years from innumerable *in vitro* and animal model studies e.g., [5-8] indicating that EMT is playing a key role in the metastatic spread of cancer cells from primary sites. Despite the significant body of support for this model, the lack of definitive clinical evidence in human cancer patients has resulted in persistent and spirited skepticism [9,10]. Much of this skepticism is based upon the fact that human metastatic samples consistently display morphologies indistinguishable from the primary tumors from which they are derived.

In an effort to help resolve this controversy, we conducted morphological and molecular analyses of 14 matched sets of primary and metastatic samples from late staged (III/IV) ovarian cancer patients. Pathological examination revealed no morphological differences between any of the primary and metastatic samples. In contrast, gene expression analyses identified two distinct groups of patient samples. In one group, the molecular profiles of primary and metastatic samples from the same patient displayed indistinguishable molecular profiles. While this result is not inconsistent with an EMT model where mesenchymal-like metastasizing cells have undergone a compensating MET transition at the metastatic site, the results are equally consistent with the hypothesis that metastasis in these patients did not involve EMT. However, molecular profiling also identified a second group of patient samples where the metastatic samples from different patients clustered together and were clearly distinct from their respective primary samples. Further analyses demonstrated that differences in the expression patterns of genes previously associated with EMT clearly separated the primary and metastatic samples isolated from all but one patient.

## Conclusions

Collectively, our results support a role of EMT in ovarian cancer metastases and demonstrate that indistinguishable morphologies between primary and metastatic cancer samples is not sufficient evidence to negate the role of EMT in the metastatic process.

### Consent

Written informed consent was obtained from the patients for the publication of this report and any accompanying images.

## Competing interests

The authors declare they have no competing interests.

## Authors’ contributions

LNL carried out data analysis and assisted in the writing of the manuscript; LVL isolated the RNA and conducted the gene expression analysis; LDW conducted the laser capture microdissection of cancer cells; SLW conducted the histological examination of samples; BBB performed the surgeries and collected tissue samples; JFM designed the study and assisted in the writing of the manuscript. All authors read and approved the final manuscript.

## Supplementary Material

Additional file 1 **Characteristics of patient samples.** Patient age and sample characteristics (histopatology, Stage, Grade and the results of a blind comparison of primary and metastatic samples by a certified pathologist. Click here for file

Additional file 2 **Expression values of all significantly differentiated genes among patient samples analyzed in this study.** Shown are gene symbols, probe set IDs and log_2_ expression values of the 4,389 probe sets used for the unsupervised clustering (Figure 1). Click here for file

Additional file 3 **Genes previously established as being implicated in epithelial-to-mesenchymal transition (EMT).** List was previously compiled by SA biosciences (http://www.sabiosciences.com/rt_pcr_product/HTML/PAHS-090Z.html). Click here for file

Additional file 4 **Expression values of EMT associated genes significantly differentiated expressed among patient samples analyzed in this study.** Gene symbols, probe set IDs, log_2_ expression values, and Z-score normalized expression values of the 61 EMT associated probe sets used for the clustering in Figure 3. Click here for file
