# Supplementary material for: Molecular profiling supports the role of epithelial-to-mesenchymal transition (EMT) in ovarian cancer metastasis
Source: J Ovarian Res. 2013 Jul 10;6:49. doi: 10.1186/1757-2215-6-49 (PMC3726281; doi:10.1186/1757-2215-6-49)
Supplement: Additional file 1 — Characteristics of patient samples. Patient age and sample characteristics (histopatology, Stage, Grade and the results of a blind comparison of primary and metastatic samples by a certified pathologist. [file 1757-2215-6-49-S1.docx]

**Additional file 1. Characteristics of patient samples.**

| **Patient ID** | **Age** | **Histopathology** | **Stage** | **Grade** | **Morphological Comparison Primary vs Metastasis** |
| --- | --- | --- | --- | --- | --- |
| 489 | 48 | Serous Adenocarcinoma | IV | 3 | Right ovary vs. Omentum: Similar morphology |
| 528 | 66 | Serous Adenocarcinoma | IIIc | 3 | Right ovary vs. Omentum: Similar morphology |
| 542 | 61 | Serous Adenocarcinoma | IV | 3 | Left ovary vs Omentum: Similar morphology |
| 551 | 59 | Serous Adenocarcinoma | IIIc/IV | 3 | Right ovary vs. omentum: Similar morphology |
| 588 | 71 | Serous Adenocarcinoma | IIIc | 2/3 | Right ovary vs. Omentum: Similar morphology |
| 617 | 64 | Serous Adenocarcinoma | IIIc | 2/3 | Left ovary vs. omentum: Similar morphology |
| 620 | 62 | Serous Adenocarcinoma | III/IV | 3 | Left ovary vs. omentum: Similar morphology |
