# Supplementary material for: Molecular profiling supports the role of epithelial-to-mesenchymal transition (EMT) in ovarian cancer metastasis
Source: J Ovarian Res. 2013 Jul 10;6:49. doi: 10.1186/1757-2215-6-49 (PMC3726281; doi:10.1186/1757-2215-6-49)
Supplement: Additional file 3 — Genes previously established as being implicated in epithelial-to-mesenchymal transition (EMT). List was previously compiled by SA biosciences (http://www.sabiosciences.com/rt_pcr_product/HTML/PAHS-090Z.html). [file 1757-2215-6-49-S3.docx]

**Additional file 3.Genes previously implicated in epithelial-to-mesenchymal transition (EMT)**

(http://www.sabiosciences.com/rt_pcr_product/HTML/PAHS-090Z.html)

**Genes Up-Regulated During EMT:** AHNAK, BMP1, CALD1, CAMK2N1, CDH2, COL1A2, COL3A1, COL5A2, FN1, FOXC2, GNG11, GSC, IGFBP4, ITGA5, ITGAV, MMP2, MMP3, MMP9, MSN, SERPINE1, SNAI1, SNAI2, SNAI3, SOX10, SPARC, STEAP1, TCF4, TIMP1, TMEFF1, TMEM132A, TWIST1, VCAN, VIM, VPS13A, WNT5A, WNT5B.

**Genes Down-Regulated During EMT:** CAV2, CDH1 (E-cadherin), DSP, FGFBP1, IL1RN, KRT19, MST1R, NUDT13, OCLN, PPPDE2, RGS2, SPP1 (Osteopontin), TFPI2, TSPAN13.

**Differentiation & Development:** AKT1, BMP1, BMP2, BMP7, COL3A1, COL5A2, CTNNB1, DSP, ERBB3, F11R, FOXC2, FZD7, GSC, JAG1, KRT14, MST1R, NODAL, NOTCH1, PTP4A1, SMAD2, SNAI1, SNAI2, SOX10, TGFB2, TGFB3, TMEFF1, TWIST1, VCAN, WNT11, WNT5A, WNT5B.

**Morphogenesis:** CTNNB1, FOXC2, JAG1, RAC1, SMAD2, SNAI1, SOX10, TGFB1, TGFB2, TGFB3, TWIST1, WNT11, WNT5A.

**Cell Growth & Proliferation:** AKT1, BMP1, BMP7, CAV2, CTNNB1, EGFR, ERBB3, FGFBP1, FOXC2, IGFBP4, ILK, JAG1, MST1R, NODAL, PDGFRB, TGFB1, TGFB2, TGFB3, TIMP1, VCAN, ZEB1.

**Migration & Motility:** CALD1, CAV2, EGFR, FN1, ITGB1, JAG1, MSN, MST1R, NODAL, PDGFRB, RAC1, STAT3, TGFB1, VIM.

**Cytoskeleton:** CAV2, KRT7, MAP1B, PLEK2, RAC1, VIM.

**Extracellular Matrix & Cell Adhesion:** BMP1, BMP7, CDH1 (E-cadherin), CDH2 (N-cadherin), COL1A2, COL3A1, COL5A2, CTNNB1, DSC2, EGFR, ERBB3, F11R, FN1, FOXC2, ILK, ITGA5, ITGAV, ITGB1, MMP2, MMP3, MMP9, PTK2, RAC1, SERPINE1 (PAI-1), SPP1 (Osteopontin), TGFB1, TGFB2, TIMP1, VCAN.

**Signaling Pathways:**
Estrogen Receptor: CAV2, ESR1 (ERa), KRT19, TGFB3. 
G-Protein Coupled Receptor: AKT1, FZD7, GNG11, RAC1, RGS2.
Integrin-Mediated: COL3A1, ILK, ITGA5, ITGAV, ITGB1, PTK2. 
Notch: FOXC2, JAG1, NOTCH1. 
Receptor Tyrosine Kinase: EGFR, ERBB3, PDGFRB, RGS2, SPARC. 
TGFß / BMP: BMP1, BMP2, BMP7, COL3A1, SMAD2, TGFB1, TGFB2, TGFB3. 
WNT: CTNNB1, FZD7, GSK3B, WNT11, WNT5A, WNT5B.

**Transcription Factors:** CTNNB1, ESR1 (ERa), FOXC2, GSC, NOTCH1, SIP1, SMAD2, SNAI2, SNAI3, SOX10, STAT3, TCF3, TCF4, TWIST1, ZEB1, ZEB2.

**Pathway source references:**

1. Collier IE, Wilhelm SM, Eisen AZ, Marmer BL, Grant GA, Seltzer JL, Kronberger A, He CS, Bauer EA, Goldberg GI. H-ras oncogene-transformed human bronchial epithelial cells (TBE-1) secrete a single metalloprotease capable of degrading basement membrane collagen.. J Biol Chem. 1988 May 15;263(14):6579-87.
2. Kaartinen V, Voncken JW, Shuler C, Warburton D, Bu D, Heisterkamp N, Groffen J. Abnormal lung development and cleft palate in mice lacking TGF-beta 3 indicates defects of epithelial-mesenchymal interaction.. Nat Genet. 1995 Dec;11(4):415-21.
3. Fernandes AM, Hamburger AW, Gerwin BI. Dominance of ErbB-1 heterodimers in lung epithelial cells overexpressing ErbB-2. Both ErbB-1 and ErbB-2 contribute significantly to tumorigenicity.. Am J Respir Cell Mol Biol. 1999 Dec;21(6):701-9.
4. Timmerman LA, Grego-Bessa J, Raya A, Bertran E, Perez-Pomares JM, Diez J, Aranda S, Palomo S, McCormick F, Izpisua-Belmonte JC, de la Pompa JL. Notch promotes epithelial-mesenchymal transition during cardiac development and oncogenic transformation.. Genes Dev. 2004 Jan 1;18(1):99-115. Epub 2003 Dec 30.
5. Baldwin BR, Timchenko NA, Zahnow CA. Epidermal growth factor receptor stimulation activates the RNA binding protein CUG-BP1 and increases expression of C/EBPbeta-LIP in mammary epithelial cells.. Mol Cell Biol. 2004 May;24(9):3682-91.
6. Huber MA, Kraut N, Beug H. Molecular requirements for epithelial-mesenchymal transition during tumor progression.. Curr Opin Cell Biol. 2005 Oct;17(5):548-58.
7. Peignon G, Thenet S, Schreider C, Fouquet S, Ribeiro A, Dussaulx E, Chambaz J, Cardot P, Pincon-Raymond M, Le Beyec J. E-cadherin-dependent transcriptional control of apolipoprotein A-IV gene expression in intestinal epithelial cells: a role for the hepatic nuclear factor 4.. J Biol Chem. 2006 Feb 10;281(6):3560-8. Epub 2005 Dec 7.
8. Lee JM, Dedhar S, Kalluri R, Thompson EW. The epithelial-mesenchymal transition: new insights in signaling, development, and disease.. J Cell Biol. 2006 Mar 27;172(7):973-81.
9. Chen Z, Li DQ, Tong L, Stewart P, Chu C, Pflugfelder SC. Targeted inhibition of p57 and p15 blocks transforming growth factor beta-inhibited proliferation of primary cultured human limbal epithelial cells.. Mol Vis. 2006 Aug 23;12:983-94.
10. Moreno-Bueno G, Cubillo E, Sarrio D, Peinado H, Rodriguez-Pinilla SM, Villa S, Bolos V, Jorda M, Fabra A, Portillo F, Palacios J, Cano A. Genetic profiling of epithelial cells expressing E-cadherin repressors reveals a distinct role for Snail, Slug, and E47 factors in epithelial-mesenchymal transition.. Cancer Res. 2006 Oct 1;66(19):9543-56.
11. Billottet C, Tuefferd M, Gentien D, Rapinat A, Thiery JP, Broet P, Jouanneau J. Modulation of several waves of gene expression during FGF-1 induced epithelial-mesenchymal transition of carcinoma cells.. J Cell Biochem. 2008 Jun 1;104(3):826-39.
12. Higashikawa K, Yoneda S, Taki M, Shigeishi H, Ono S, Tobiume K, Kamata N. Gene expression profiling to identify genes associated with high-invasiveness in human squamous cell carcinoma with epithelial-to-mesenchymal transition.. Cancer Lett. 2008 Jun 18;264(2):256-64. Epub 2008 Mar 10.
13. Lavery K, Swain P, Falb D, Alaoui-Ismaili MH. BMP-2/4 and BMP-6/7 differentially utilize cell surface receptors to induce osteoblastic differentiation of human bone marrow-derived mesenchymal stem cells.. J Biol Chem. 2008 Jul 25;283(30):20948-58. Epub 2008 Apr 24.
14. Yang J, Weinberg RA. Epithelial-mesenchymal transition: at the crossroads of development and tumor metastasis.. Dev Cell. 2008 Jun;14(6):818-29.
15. Fournier HN, Dupe-Manet S, Bouvard D, Lacombe ML, Marie C, Block MR, Albiges-Rizo C. Integrin cytoplasmic domain-associated protein 1alpha (ICAP-1alpha ) interacts directly with the metastasis suppressor nm23-H2, and both proteins are targeted to newly formed cell adhesion sites upon integrin engagement.. J Biol Chem. 2002 Jun 7;277(23):20895-902. Epub 2002 Mar 27.
